# Mechanical Characteristics and Skating Performance of Trained Youth Ice Hockey Players at Different Maturation Stages

**DOI:** 10.3390/jfmk11010002

**Published:** 2025-12-21

**Authors:** Julien Glaude-Roy, Jean Lemoyne

**Affiliations:** 1 Department of Human Kinetics, Université du Québec à Trois-Rivières, Trois-Rivières, QC G8Z 4M3, Canada; julien.glaude-roy@uqtr.ca; 2 Laboratoire de Recherche sur le Hockey UQTR Score, Université du Québec à Trois-Rivières, Trois-Rivières, QC G8Z 4M3, Canada

**Keywords:** acceleration, speed, puberty, force–velocity, biomechanics, testing

## Abstract

**Objectives:** This study aimed to investigate the skating force–velocity (F–V) mechanical characteristics of trained youth ice hockey players at different stages of their maturational development. **Methods:** A total of 52 male trained ice hockey players (14.6 ± 1.4 years) from U13, U15, U17, and U18 competitive teams of the same hockey program were classified into three maturation groups—Pre-, Mid-, and Post-peak height velocity (PHV). Participants performed two 40 m maximal skating efforts while velocity data were collected using a radar device to derive F–V parameters (e.g., theoretical maximal force (F_0_), velocity (V_0_), power (P_max_), and related metrics). The maturation offset was computed using the following formula: Maturity offset = −8.128741 + (0.0070346 · (Chronological age · Sitting height)). **Results:** Results revealed significant effects of puberty on most performance variables (F_(2,49)_ = [5.58, 31.72]; *p* ≤ 0.07; η^2^ = [0.19, 0.56]). Differences in acceleration (0–10 m time) and F_0_ improved markedly between Mid- and Post-PHV stages (|d| = [1.38, 1.92]), while V0 and maximal sprint velocity (30–40 m time) improved constantly across maturation stages (|d| = [1.03, 1.99]). **Conclusions:** This is the first study to provide reference skating F–V profile values across puberty in trained youth male ice hockey players. Coaches and practitioners are encouraged to prioritize acceleration and skating technique early during puberty to maximize velocity development and emphasize strength development after reaching peak height velocity. Conclusions should be considered with care as the Pre-PHV group was small (*n* = 5) and the used F–V method remains to be validated on ice.

## 1. Introduction

Maximal forward skating ability constitutes a primary determinant of ice hockey performance. During competition, players typically spend 17.6% of effective playing time at velocities exceeding 22 km·h^−1^ [1], covering between 300 and 900 m above 24 km·h^−1^ [2]. Consequently, the capacity to achieve and sustain high levels of speed and acceleration is essential for optimal on-ice performance. The development and maintenance of maximal forward skating performance represent a major focus for strength and conditioning professionals. Selecting the most appropriate training intervention for a given athlete remains challenging, as various methods—including Olympic weightlifting, plyometric training, traditional strength training, and resisted sprinting—have each demonstrated potential to enhance maximal skating speed [3,4,5,6]. This issue is salient among adolescent players, given the extensive physiological and morphological adaptations associated with growth and maturation. Increases in muscle mass, stature, and circulating testosterone levels enhance force and power production capacities, thereby improving overall physical performance [7]. However, existing evidence suggests that the efficacy of specific training modalities may vary considerably according to the athlete’s maturation status and individual responsiveness [7,8,9].

Samozino et al. [10] introduced a practical field method for assessing sprint performance through the analysis of an athlete’s horizontal force–velocity (F–V) profile. Using a smartphone-based application, practitioners can easily obtain detailed insights into an athlete’s sprint mechanical capacities, making this approach highly accessible—particularly for adolescent athletes. The horizontal F–V profile enables the estimation of ground reaction forces across the entire acceleration phase from displacement or velocity data [11]. From these estimations, several key mechanical variables can be derived, including maximal theoretical force relative to body mass (F_0_), maximal theoretical velocity (V_0_), maximal theoretical power relative to body mass (P_max_), the force–velocity slope (S_fv_), the maximal ratio of the horizontal component of the ground reaction force (RF_max_), and the rate of decrease in this ratio with increasing velocity (D_rf_) [10]. The slope of the F–V relationship (S_fv_) can then be compared with an “optimal” slope (S_fvopt_) to guide individualized training prescriptions [12], allowing practitioners to identify whether an athlete presents a force or velocity deficit. Given its practicality and the importance of maximal forward skating in ice hockey, horizontal F–V profiling offers a valuable and accessible tool for individualized performance training in applied sport settings.

To our knowledge, no study has verified the validity of the F–V method in skating. Yet, Perez et al. [13] and Stenroth et al. [14] have demonstrated the reliability of F–V profiling for the analysis of skating sprints. Data specific to ice hockey players are very limited with less than 10 studies on the topic. Among the few studies, Perez et al. [15] explored associations between the F–V characteristics in sprinting, skating, and jumping of 17 female players from the French national team. Results highlighted that P_max_ displayed the strongest associations across testing modalities while other variables only exhibited small associations between sprinting and skating. Since physical maturation can influence attributes such as speed and power, it is important to consider its potential role in skating performance. In this regard, only two studies specifically investigated ice hockey players’ F–V during puberty [16,17]. Recently, Glaude-Roy et al. [16] explored associations between the sprinting F–V, repeated sprint ability, and anaerobic capacity of 70 highly trained, adolescent ice hockey players. They compared the associations between the running F–V profile, a repeated sprint test, and the 30 s Wingate test. Moderate-to-large correlations were observed between P_max_, V_0_, and both anaerobic tests, while small-to-moderate correlations were found between F_0_ and the anaerobic tests. These findings highlight the importance of developing a solid general physical foundation in adolescent players. In a second study, Glaude-Roy et al. [17] explored the associations between skating F–V and 30 m running sprint time, change in direction ability in a 5-10-5 test, and lower limb explosive strength, measured by distance in the standing long jump of 107 highly trained adolescent ice hockey players. Correlation analysis revealed moderate associations between off-ice tests and F_0_ or V_0_ and moderate-to-large associations with P_max_. Multivariate analysis of covariance for F_0_, V_0_, and off-ice tests suggested that change in direction and linear speed training may have distinct impacts on the development of F_0_ and V_0_. Again, the results stressed the importance of prioritizing a good general physical preparation at this stage of athletic development (e.g., puberty).

Since these studies were conducted on homogenous groups in terms of expertise level and physical maturation, the specific impacts of biological age on ice hockey players’ skating performance remain unclear. Refining our understanding of these associations is particularly important for youth players who, at puberty, go through a maturation process characterized by a growth spurt and increase in muscle mass associated with higher strength and power capacities [7]. In this regard, adolescent athletes might display large differences in terms of physical maturation, leading to discrepancies in terms of physical performance [18]. Because of this, understanding how mechanical components of the F–V differ at different maturational stages is crucial. Fernández-Galván et al. [9] have explored the changes in sprint F–V mechanics of youth soccer players. They measured the sprint F–V of 62 players between 10 and 18 years old while estimating their maturational stage from age and sitting height. They observed distinct differences between force and velocity according to the maturational stage of players determined by peak height velocity (PHV). Specifically, large to very large effects were observed in early adolescence (Pre-to-Mid-PHV) for strength- and acceleration-related variables while velocity-related variables showed larger effects later in adolescence (Mid-to-Post-PHV). Skating is a unique skill with clear distinctions from sprinting. Skating can be decomposed into a running-like phase in the first three to seven steps and a gliding phase. In contrast, sprinting shows a gradual change in the tronc’s forward incline [19]. There are also clear differences in hip, knee, and ankle kinematics where skating requires more lateral movements [19]. Despite important differences in skating mechanics between high-caliber and recreational adult ice hockey players [20], no research has focused on the skating mechanical capabilities of adolescent ice hockey players during puberty. Therefore, the aim of this study was to investigate differences in skating performance and force–velocity (F–V) mechanical characteristics among trained youth ice hockey players at various maturational stages. In line with the findings of Fernández-Galván et al. [9] in soccer players, we assumed that mechanical variables associated with force production (e.g., acceleration and F_0_) would be higher after puberty and show larger differences between Pre- and Mid-PHV. Variables related to velocity (e.g., maximal speed and V_0_) would also be higher after puberty with larger differences between Mid- and Post-PHV.

## 2. Materials and Methods

### 2.1. Participants and Study Design

In this retrospective study, 80 players (20 per team) from the same hockey program in the province of Quebec were invited to participate. The players belonged to the under-13, under-15, under-17, and under-18 competitive teams (U13, U15, U17, and U18, respectively). Injured players (*n* = 4) and players absent on testing day (*n* = 16) were not tested. Goaltenders (*n* = 8) were removed due to the specific nature of their skating demands. Ultimately, the force–velocity (F–V) mechanical characteristics and maximal skating times were analyzed for 52 male ice hockey players. An a priori power analysis conducted in G*Power 3.1.9.7 for fixed effects in a one-way ANOVA with three groups indicated that the sample size across the three maturational subgroups was adequate (α = 0.05; power = 0.80; effect size = 0.50). Testing took place mid-season, during the last week of November and the first week of December 2024, at the beginning of the players’ regular on-ice training sessions (first day of the week). The most recent match had been played at least 48 h before testing. All participants were classified as trained athletes, as they practiced four times per week and competed at the provincial level [21]. All athletes who took part in this study were supervised by a strength and conditioning coach (assigned to the hockey program) throughout their season. For players under 14 years of age, parents were informed of this study’s purpose, and they provided written consent prior to participation. Written informed consent was also obtained from all players and from the parents (e.g., if <14 years old). All procedures complied with provincial regulations governing research with human participants in Quebec, Canada. The study protocol was approved by the institutional research ethics committee (CER-25-320-07.10).

### 2.2. Variables

#### 2.2.1. Skating Performance

Players performed two 40 m maximal skating efforts with 5 min rest between trials. This distance was based on Stastny et al.’s [22] recommendations to reach maximal velocity while skating. Prior to testing, players performed a 10 min general warm-up combining general skating around the ice, specific skating drills, edge work, and short accelerations. Skating times were measured using Swift single-beam laser gates (Swift Performance, Northbrook, IL, USA) placed one meter above ground at 0, 10, 30, and 40 m. This method was inspired by Bundle et al. [23] who used the last meters of an overground sprint to estimate maximal speed. A starting line was drawn 50 cm behind the first gate to minimize variation between trials due to variability in triggering patterns with such systems [22]. Players were asked to hold a static crouched position on the line while facing forward with their stick on the ice. When the system was ready, a previously trained research assistant allowed players to start skating. They were also instructed to give their maximal effort during both trials. Such testing methods are reliable when the intra-class correlation coefficient (ICC) is >75% and the coefficient of variation (CV) is <5% [24,25]. The fastest times to complete the first and last 10 m were used for analysis and considered as acceleration (0–10 m time) and maximal velocity (30–40 m time). The ICC for skating times were 96% and 75%, respectively.

Simultaneously, a Stalker ATS II radar (Stalker Sport, Richardson, TX, USA) was placed three m behind the starting line and one meter above ground approximately at the height of players’ center of mass to follow instantaneous velocity at 48.875 Hz. In contexts that are specific to ice hockey, the radar protocol displayed acceptable reliability, with an ICC of >75% and a CV of <10% for all F–V variables [13]. Following these testing procedures, raw velocity data were first imported in R studio and treated following previous research designs [10,13]. Then, data prior to the skating start and after the maximal velocity plateau were discarded. Mechanical variables were calculated after the remaining data were fitted with a mono-exponential function [10,13]. This approach was shown to be valid and reliable for testing team-sport athletes [26]. The average values of F–V variables for both trials were used for group comparisons [26].

#### 2.2.2. Physical Maturation

Maturity offset (MO) was assessed by using the updated equation from Moore et al. [27], which refers to the distance in years from age at PHV:

(1) Maturity offset (MO) = −8.128741 + (0.0070346 × [Chronological age × Sitting height]).

Once the maturity offset (MO) was calculated, participants were categorized into one of three maturational groups: (1) Pre-PHV, (2) Mid-PHV, or (3) Post-PHV. The classification criteria were defined as follows: (1) MO < −1 for Pre-PHV; (2) −1 ≤ MO ≤ 1 for Mid-PHV; and (3) MO > 1 for Post-PHV. This computational method provides a valid estimate of the actual age at PHV [28].

### 2.3. Statistical Analyses

Normality assumptions were verified with the Shapiro–Wilk test showing a violation for 30–40 m times (SW = 0.858, *p* < 0.001). Further investigation showed acceptable scores of skewness and kurtosis (skewness = 1.0, SE = 0.33; kurtosis = −0.076, SE = 0.65) and showed no major violations of normality; thus, the 30–40 m times were included in the analysis. Differences between maturation stages (e.g., Pre-PHV, Mid-PHV, and Post-PHV) for skating F–V variables and skating times were determined using one-way ANOVAs. To prevent bias due to violation of normality and unequal samples (e.g., Pre-PHV), a bootstrapping approach (5000 resamples with percentile method) was used to minimize bias of estimation and to provide adjusted confidence intervals [29]. Following this procedure, post hoc analysis using Fisher’s LSD was conducted to identify specific group differences. Eta-squared effect sizes (η^2^) for the omnibus ANOVA were performed, whereas Cohen’s D was computed to estimate post hoc effect sizes and were interpreted as trivial <0.2; small = 0.2–0.6; moderate = 0.6–1.2; large = 1.2–2.0; very large = 2.0–4.0; and extremely large >4.0 [30]. Statistical analyses were conducted using SPSS version 28.0 (IBM Corporation, Armonk, NY, USA).

## 3. Results

The descriptive statistics of skating times and the skating F–V variables according to the maturational stage are presented in Table 1. Age corresponds to the age group categories in which players evolve. In terms of the proportion of players according to their maturational stage, the Pre-PHV group was underrepresented (10% of the total sample) and had members of the younger team (U13). The Mid-PHV group was the most represented (54%), whereas the Post-PHV group consisted of 36% of the total sample (all of them being in the U17–U18 team), which is congruent with the maturation processes during adolescence. Table 1 also shows specific results according to each F–V variable.

**Table 1:** Descriptive statistics (mean ± standard deviation) for all variables calculated using bootstrap analysis.

	Pre-PHV *n* = 5 (10%)	Mid-PHV *n* = 28 (54%)	Post-PHV *n* = 19 (36%)
Age (years)	12.8 ± 0.5 [12.2, 13.4]	14.0 ± 1.0 [13.6, 14.4]	16.0 ± 0.7 [15.7, 16.3]
Stature (m)	1.48 ± 0.09 [1.36, 1.59]	1.69 ± 0.06 [1.66, 1.71]	1.72 ± 0.05 [1.70, 1.74]
Sitting height (m)	0.75 ± 0.05 [0.68, 0.82]	0.84 ± 0.04 [0.82, 0.85]	0.87 ± 0.02 [0.86, 0.88]
Mass (kg)	40.6 ± 3.0 [36.9, 44.3]	60.2 ± 9.1 [56.7, 63.7]	72.6 ± 4.6 [70.4, 74.8]
Maturity offset (years)	−1.38 ± 0.37 [−1.84, −0.93]	0.10 ± 0.58 [−0.13, 0.32]	1.63 ± 0.37 [1.45, 1.81]
0–10 m time (s)	2.20 ± 0.09 [2.10, 2.27]	2.11 ± 0.16 [2.05, 2.17]	1.84 ± 0.11 [1.79, 1.89]
30–40 m time (s)	1.39 ± 0.13 [1.26, 1.48]	1.22 ± 0.13 [1.17, 1.27]	1.07 ± 0.05 [1.05, 1.09]
F_0_ (N·kg^−1^)	4.43 ± 0.52 [4.02, 4.98]	4.71 ± 0.56 [4.51, 4.92]	5.44 ± 0.48 [5.22, 5.64]
V_0_ (m·s^−1^)	8.60 ± 0.37 [8.32, 8.97]	9.20 ± 0.61 [8.98, 9.42]	10.32 ± 0.48 [10.11, 10.54]
P_max_ (W·kg^−1^)	9.53 ± 1.25 [8.50, 10.97]	10.87 ± 1.89 [10.20, 11.60]	14.03 ± 1.48 [13.36, 14.69]
S_fv_ (N·m·s^−1^)	−0.52 ± 0.06 [−0.58, −0.48]	−0.51 ± 0.05 [−0.53, −0.49]	−0.53 ± 0.05 [−0.55, −0.51]
RF_max_ (%)	31.6 ± 4.35 [27.94, 35.81]	34.15 ± 3.9 [32.67, 35.64]	36.96 ± 3.06 [35.57, 38.25]
D_rf_ (%)	−5.0 ± 0.56 [−5.54, −4.59]	−4.91 ± 0.4 [−5.07, −4.75]	−5.10 ± 0.43 [−5.19, −4.82]

Results are reported with 95% confidence intervals (in brackets). F_0_: maximal theoretical force; V_0_: maximal theoretical velocity; P_max_: maximal theoretical power; S_fv_: force–velocity slope; RF_max_: the maximal ratio of the horizontal component of the ground reaction force; D_rf_: the rate of decrease in the ratio of the horizontal component of the ground reaction force.

**Figure 1:**
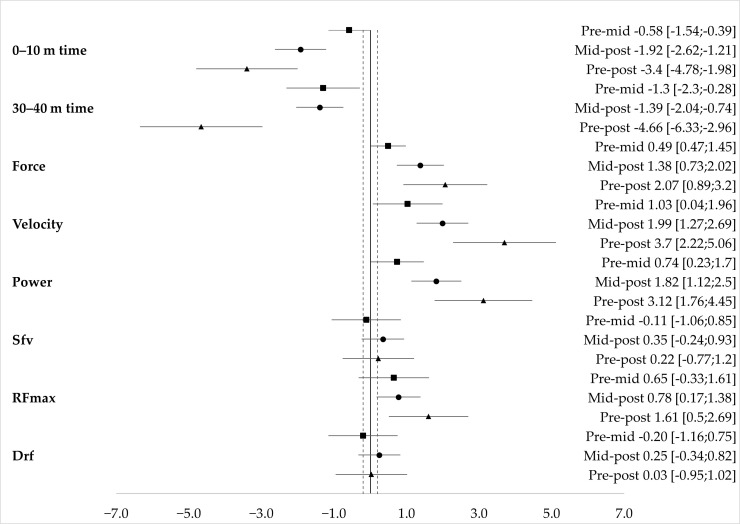
Post hoc effect sizes with 95% confidence intervals of differences according to different maturation statuses.

Results of the one-way bootstrapped ANOVA are presented in Table 2. A posteriori statistical power was satisfactory for all tests (*p* > 0.90), except for D_rf_ and S_fv_ (*p* = 0.11). There were significant differences between maturational stages for all variables, with the exception of S_fv_ and D_rf_. Effect sizes from post hoc analysis with 95% confidence intervals for skating times and skating F–V variables from Pre-to-Mid-PHV (pre-mid), Mid-to-Post-PHV (mid-post), and Pre-to-Post-PHV (pre-post) are presented in Figure 1.

**Table 2:** Impact of physical maturation on skating performance: group differences (from bootstrapped ANOVAs) in acceleration time, maximal speed time, and F–V variables.

	F_(2,49)_	*p*	η^2^
0–10 m time (s)	26.61	<0.001	0.52 [0.30, 0.64]
30–40 m time (s)	20.86	<0.001	0.46 [0.23, 0.59]
F_0_ (N·kg^−1^)	13.44	<0.001	0.35 [0.13, 0.51]
V_0_ (m·s^−1^)	31.72	<0.001	0.56 [0.35, 0.68]
P_max_ (W·kg^−1^)	24.83	<0.001	0.50 [0.28, 0.63]
S_fv_ (N·m·s^−1^)	0.64	0.53	0.03 [0.00, 0.13]
RF_max_ (%)	5.58	0.007	0.19 [0.02, 0.35]
D_rf_ (%)	0.35	0.71	0.01 [0.00, 0.10]

F_0_: maximal theoretical force; V_0_: maximal theoretical velocity; P_max_: maximal theoretical power; S_fv_: force–velocity slope; RF_max_: the maximal ratio of the horizontal component of the ground reaction force; D_rf_: the rate of decrease in the ratio of the horizontal component of the ground reaction force.

## 4. Discussion

This study investigated differences in skating times and force–velocity (F–V) mechanical characteristics among trained youth ice hockey players across distinct maturational stages. The main effect sizes for comparisons between Pre-PHV and Mid-PHV, as well as between Mid-PHV and Post-PHV, demonstrated greater differences in acceleration-related variables (F_0_ and 0–10 m time) during the later stages of maturation. This observation presumably indicates that acceleration variables are developed after PHV. In contrast, velocity-related variables (V_0_ and 30–40 m time) showed consistently higher values across maturational stages, in favor of the Post-PHV group. These results are somewhat in contradiction with the initial hypothesis. Fernández-Galván et al. [9] observed large differences between the Pre- and Mid-PHV youth soccer player groups for acceleration-related variables and large differences between Mid- and Post-PHV for velocity-related variables. While they represent maximal physiological abilities of athletes, maximal forward skating and sprinting are very different biomechanically. In skating, there is a clear change between a more running-like motion during the acceleration phase to a gliding phase after four to seven steps [19]. Recent studies conducted by Kaartinen et al. [31,32] showed that muscular activity patterns analyzed during 30 m skating tests (e.g., swing, propulsion, recovery) tend to vary slightly through the skating cycle. This suggests that skating efficiency might be a key factor in skating F–V characteristics. As skating efficiency has been identified as an important determinant of the cardiorespiratory fitness of hockey players [33], it should be considered in future studies examining the skating speed and acceleration performance of hockey players. In line with these observations, ice friction may also explain a good part of the differences in F–V between skating and sprinting. During the first steps, low ice friction causes poor blade-to-ice traction, thus minimizing force application during acceleration [19]. At maximal velocity, the low ice friction helps skaters reach higher velocities by increasing gliding distances. After the running phase, the crouched positing used to maximize lateral pushes with the blade and glide efficiently lead to large changes in the center of mass acceleration between each step [34]. In sum, the way players accelerate forward on the ice and glide at top speed are depicted by lower acceleration values and higher velocity values when compared to sprinting [16].

While previous studies have reported values for highly competitive male and female players [13,16,17], this study is the first to report the skating F–V of an adolescent population of competitive ice hockey players. The Mid-PHV group exhibited similar F–V capabilities than the highly competitive players in previous studies (for example, F_0_: 4.65 vs. 4.71 or V_0_: 9.83 vs. 9.20). This may be explained by the very similar chronological ages of players in both studies (14.0 vs. 13.8) and a large group of players with similar physical abilities in the province of Quebec. For example, Lemoyne et al. [35] showed that a global physical testing protocol might not be sufficient to explain the selection process of 40 male players (from an initial pool of 80 athletes) invited to the provincial development camp. Players were selected from an approximate pool of 450 highly trained players between 15 and 18 years of age identified by the federation. It went on to show the very competitive field of youth male players in the province. This may explain why the Mid-PHV players from this study have very similar skating F–V mechanical characteristics to those previously reported even though they are not part of the selection process of the federation. Nonetheless, the study results expand the current knowledge regarding the skating F–V of adolescent ice hockey players as it gives a complete overview of how it evolves during puberty. The values can guide practitioners in setting training goals specific to the players’ development or maturation stage for an individualized training prescription (see Hicks et al. [36] for the practical application of this approach). Practitioners are encouraged to prioritize acceleration-focused drills during early puberty and focus on resisted skating once players have reached PHV.

While this study adds to the current knowledge, some limitations should be considered. The first limitation concerns the selection bias related to physical maturation. As mentioned earlier, Pre-PHV players were underrepresented in this study sample (10% of the total sample). This imbalance may have reduced the robustness of group comparisons and limited the generalizability of the findings by increasing the likelihood of type II errors. However, it should be noted that a similar imbalance has been reported in previous studies involving adolescent hockey players. In those studies, early-maturing players were clearly overrepresented, as they are often selected at the early stages of development [37,38]. To overcome this limitation, future research should consider adjusting the sampling strategy to increase the proportion of late-maturing players, particularly by including such participants in each age-group category. As the growth spurt can begin at 12 years of age for males [7], a portion of the U13 players were in the beginning of their maturation process, placing them in the Mid-PHV group. In comparison, European soccer clubs are managed outside of the school system, which allowed Fernández-Galván et al. [9] to recruit participants in younger teams. To improve the robustness and generalizability of the study results, the participation of players from a U11 team is encouraged. The second limitation concerns the research design. Since this study used a retrospective cross-sectional design, data were structured into different categories (e.g., Pre-, Mid-, and Post-PHV, which were independent of each other). This limits the direct observation of how F–V variables evolve during puberty. To completely understand the changes in skating F–V mechanical characteristics during puberty, more longitudinal studies are recommended [39]. Drawing individual curves for changes in the F–V during maturation would confirm or disapprove the study observations. The third limitation to this study is about skating kinematics. Previous studies with adult players showed significant kinematic differences between high- and low-caliber players [20]. Tracking these variables concurrently for skating F–V should allow a further understanding of links with skating mechanical capabilities and maturation. Finally, the skating F–V measures used during this study have gone through reliability analysis for ice hockey [13,14], but their validity is yet to be confirmed. In its initial stages of development, the sprinting F–V was validated by using step average values of the ground reaction force on a specialized treadmill [40]. The large movement of the trunk during the running-like motion of the first steps of maximal skating efforts could lead to an underestimation of acceleration metrics (e.g., F_0_ and Rf_max_). Moreover, this method omits ice friction and only considers air friction that may also lead to an underestimation of these parameters. Using plantar insoles to measure each stride [41] could confirm the radar’s validity.

## 5. Conclusions

This study examines in detail the skating force–velocity mechanical capabilities among trained youth ice hockey players at three stages of maturation. The results suggest substantial differences in acceleration-related variables during the later stages of maturation, whereas differences in velocity-related variables were consistent between stages. These results provide practitioners (e.g., strength and conditioning coaches) with valuable reference data to support individualized training prescriptions for competitive adolescent hockey players. Emphasis should be placed on acceleration-oriented drills and skating techniques during the early stages of maturation, while the development of muscular strength—through resisted skating or strength training—becomes increasingly important after puberty. These conclusions should be considered with caution. The small sample size for the Pre-PHV and the questionable validity of the F–V method for skating should be considered in future research.

## Data Availability

Raw data supporting the conclusions of this article is available as Supplementary File.

## Supplementary Materials

The following supporting information can be downloaded at: https://www.mdpi.com/article/10.3390/jfmk11010002/s1.

## Abbreviations

The following abbreviations are used in this manuscript:

CV	Coefficient of variation
D_rf_	Rate of decrease in the ratio of the horizontal component of the ground reaction force
F–V	Force–velocity profile
F_0_	Maximal theoretical force
PHV	Peak height velocity
P_max_	Maximal theoretical power
RF_max_	Maximal ratio of the horizontal component of the ground reaction force
S_fv_	Force–velocity slope
V_0_	Maximal theoretical velocity
